# Age-sex disparities and sub-clinical hypothyroidism among patients in Tikur Anbesa Specialized Hospital, Addis Ababa, Ethiopia

**DOI:** 10.1186/s41043-018-0149-x

**Published:** 2018-07-20

**Authors:** Habtamu Azene Tekle, Tesfahun Molla Bobe, Efrata Girma Tufa, Fithamlak Bisetegen Solomon

**Affiliations:** 1 College of Health Science and Medicine, School of Medicine, Wolaita Sodo University, Wolaita Sodo, Ethiopia; 2 College of Health Science and Medicine, School of Public Health, Wolaita Sodo University, Wolaita Sodo, Ethiopia; 3 Department of Medical Laboratory, College of Health Science and Medicine, Wolaita Sodo University, PO Box 138, Wolaita Sodo, Ethiopia

**Keywords:** Subclinical-hypothyroidism, Age, Sex, TT4, TSH, TT3, TASH

## Abstract

**Background:**

Subclinical hypothyroidism is an elevation in serum thyroid-stimulating hormone above the upper limit of the reference range (0.45–4.5 mIU/L) with normal serum TT4 and TT3 concentration. The most important implication of subclinical hypothyroidism is high likelihood of progression of clinical hypothyroidism.

**Methods:**

Institution-based cross-sectional study was conducted on medical records of patients referred at endocrine clinic Tikur Anbesa Specialized Hospital, Addis Ababa from 2010 to 2016. This study was conducted from normal ambulatory patients who have come in the hospital outpatient department since they experienced abnormality on their health status. During the study period, patients were complaining about their clinical symptoms. A total number of 9000 patients were included. Patients’ card was retrieved by using standard extracted formats to collect socio-demographic and clinical information and laboratory measurements. Serum TSH, TT4, and TT3 levels were determined by electro-chemiluminescence immunoassay method on ECLIA 2010 fully automatic analyzer at TASH nuclear medicine. SPSS 20 version software was used for analysis, and chi-square test was used to check the association between dependent and independent variables.

**Results:**

The overall prevalence of subclinical hypothyroidism evaluated to be 582 (6.47%), 4.6% in females and 1.9% in males. Four hundred and thirty-one (74%) patients had serum TSH levels between 5 and 10 mIU/L, and the average TSH level of subclinical hypothyroid patients whose age was ≥ 40 differ significantly from that of subclinical hypothyroid patients whose age was < 40. The average TSH level among female patients whose age are ≥ 40 differed significantly from their counterparts. Subclinical hypothyroidism patients more often reported having dry skin, poor memory, fatigue, cold intolerance, constipation, and hoarseness.

**Conclusion:**

The overall prevalence of ScHt was 6.5% where females showed higher level than males. Age ≥ 40 became independent factor of subclinical hypothyroidism. The higher prevalence of subclinical hypothyroidism in this study could become a predictor for overt hypothyroidism, so screening for subclinical hypothyroidism prevents the later development of complicated overt hypothyroidism.

## Background

Sub-clinical hypothyroidism (ScHt) is a commonly encountered laboratory finding in clinical practice, defined biochemically as the finding of a raised serum thyroid-stimulating hormone (TSH ≥ 4.5 mIU/L) above the reference range from normal circulating levels of total thyroxine (T_4_) and triiodothyronine (T_3_) (TT4 = 62–141 nmol/L and TT3 = 1.1–3.2 nmol/L). Serum TSH is raised in primary hypothyroidism in which T_3_ and T_4_ are low, but in mild hypothyroidism, T_4_ may be normal but TSH will be raised [1, 2].

Subclinical hypothyroidism is the most prevalent thyroid disorder affecting 3–12% of the adult population [3–11], and the incidence of subclinical thyroid diseases or their progression is baseline TSH level, old age, female sex, and the presence of thyroid autoantibody [12].

Due to apparently asymptomatic nature of the illness, the “American Thyroid Association” has recommended routine population screening for both sexes at age 35 years and then every 5 year thereafter for early detection and treatment of ScHt [3]. Higher prevalence was observed in older populations in epidemiological surveys conducted so far [13–18]. The highest age and sex-specific rates were in women older than 60 years of age [8, 14]. Its prevalence among men over the age of 74 years (16%) was almost as high as it was in women of the same age (21%) [8].

In the Whickham survey, a large, good-quality, population-based study with 20-year follow-up, prevalence was 4 to 5% among women aged 18 to 44, 8 to 10% among women aged 45 to 74, and 17.4% among women older than age 75. The prevalence was 1 to 3% among men aged 18 to 65 and 6.2% among men over age 65 [19].

Complications associated with subclinical hypothyroidism are less known than overt hypothyroidism though there is a great body of evidence that suggests negative effects of elevated TSH on health like cardiac dysfunction, higher low-density lipoprotein cholesterol, depression, cognitive dysfunction, neuromuscular and neuropsychiatric symptoms, and decrease in quality of life [12, 20, 21].

Subclinical thyroid dysfunction, which can be diagnosed by thyroid function tests before symptoms and complications occur, is viewed as a risk factor of developing hyperthyroidism and hypothyroidism complications. The goal of screening is to identify and treat patients with subclinical thyroid dysfunction before they develop these complications [21].

A large-scale survey of the prevalence of ScHt has not been performed in Ethiopia. Since no data is available on ScHt, this study provides a preliminary understanding of magnitude of ScHt and age and sex impact on its level of [22].

## Methods

### Study area

The study was conducted in the Nuclear Medicine Center of Tikur Anbesa Specialized Hospital (TASH), Addis Ababa. This hospital serves as a referral hospital for heterogeneous group of patients from different regions of the country for thyroid function test. Tikur Anbesa Specialized Hospital was the pioneer and the largest hospital in the country. TASH is the only hospital in the country where it has endocrinologist, endocrine clinic, and nuclear medicine laboratory during the study periods. Study subjects in the study did not report any underlying disease, and all of the study participants were visiting the outpatient department for health checkup.

### Study design and period

A retrospective cross-sectional study was conducted among patients who came to the medical ward suspected of having thyroid problem of TASH in Addis Ababa from July 2, 2010, to October 17, 2016.

### Source population

All patients who came to TASH medical ward clinic during the study period are the source population of this study.

### Study population

All patients who came to TASH fulfill the inclusion criteria and selected to participate in the study.

### Inclusion criteria

The inclusion criteria of the study are adults whose age group is ≥ 18.

### Exclusion criteria

The exclusion criteria of the study are the following:

- ✓ Pregnant women
- ✓ Patients with previous diagnosis of thyroiditis
- ✓ Treated Graves’ disease or toxic multinodular goiter
- ✓ Patients undergoing thyroid surgery
- ✓ Alcoholics
- ✓ Smokers
- ✓ Patients taking thyroid medications

### Sample size determination

A total number of 9456 patients were visiting the outpatient department, endocrine clinic between the years 2010 to 2016. Four hundred fifty-six patients were excluded from the study due to age criteria and incomplete information.

### Data collection

All necessary information was collected from patient’s medical record coming to the hospital for the past 6 years (2010–2016) using structured formats. The data about demographic characteristics, clinical findings, and laboratory investigations was collected from all the sampled individuals. All the data collection formats was filled in nuclear medicine center of TASH by five clinical nurses. Clinical symptoms were collected from patient cards based on symptoms like depression, fatigue, hyperlipidaemia, hyper-homocysteinaemia, goiter, coarse hair, cold intolerance, constipation and weight gain, hoarseness, hearing loss, menorrhagia, slow return phase in knee reflexes, bradycardia, coronary artery disease, or cardiac risk factors. The sources of the information were secondary data gathered from patients chart and laboratory data. The authors of this study were part of the patient investigation team. Both subjective and objective assessment technique was used by using clinical history, physical examination, and laboratory investigation.

### Blood sample

Between 3 and 5 ml of blood was drawn from ante-cubital vein of the arm by trained laboratory technicians working at Nuclear Medicine Center. The serum was separated after 45 min of collection by centrifugation at 3500 rpm for 15 min. Then, the serum was collected in a nunc tube and stored in refrigerator at − 20 °C until analysis.

### Biochemical analysis of laboratory variables

TSH, TT4, and TT3 were measured using Elecsys 2010 immunoassay analyzer which is a fully automatic run-oriented analyzer system for the determination of immunological tests using the electro-chemiluminescence immunoassay “ECLIA” process. All components and reagents for routine analysis are integrated in or on the analyzer.

### TSH assay

Sandwich immunoassay with total duration of assay of 18 min was used. It has two-step incubation periods. During the first incubation period 50 μL of sample, a biotinylated monoclonal TSH-specific antibody and a monoclonal TSH-specific antibody labeled with a ruthenium complex reacts to form a sandwich complex. At the second incubation, streptavidin-coated micro-particles were added and the complex becomes bound to the solid phase via interaction of 20 biotin and streptavidin. The reaction mixture was aspirated into the measuring cell where the microparticles are magnetically captured onto the surface of the electrode. Unbound substances then removed with ProCell/ProCell M. Application of a voltage to the electrode induces chemiluminescent emission which was measured by a photomultiplier.

### TT3 assay

Competitive immunoassay system with total duration of assay of 18 min was used. It has two-step incubation periods. During the first incubation period, 15 μL of sample and an anti-T3-specific antibody labeled with a ruthenium complex was mixed and the second incubation period starts with addition of biotinylated T3 and streptavidin-coated microparticles, and the still-free binding sites of the labeled antibody become occupied, with formation of an antibody-hapten complex. The entire complex bounds to the solid phase via interaction of biotin and streptavidin. The reaction mixture then aspirated into the measuring cell where the microparticles are magnetically captured onto the surface of the electrode. Unbound substances were removed with ProCell/ProCell M. Application of a voltage to the electrode then induces chemiluminescent emission which was measured by a photomultiplier.

### TT4 assay

Competition immunoassay system with total duration of assay of 18 min was used. It has two-step incubation periods. During the first incubation period, 15 μL of sample and an anti-T4-specific antibody labeled with a ruthenium complex was mixed. And the second incubation period starts with addition of biotinylated T4 and streptavidin-coated microparticles, and the still-free binding sites of the labeled antibody become occupied with formation of an antibody-hapten complex. The entire complex was bounded to the solid phase via interaction of biotin and streptavidin. The reaction mixture then aspirated into the measuring cell where the microparticles are magnetically captured onto the surface of the electrode. Unbound substances were removed with ProCell/ProCell M. Application of a voltage to the electrode then induces chemiluminescent emission which was measured by a photomultiplier.

Results of all of the hormones of thyroid functions test TSH, TT4, and TT3 were determined via a calibration curve which is instrument-specifically generated by 2-point calibration and a master-curve provided via the reagent barcode.

### Ethical consideration

This study was approved by the Institutional Review Board (IRB) of the School of Medicine, Addis Ababa University on January 1, 2010. Permission letter was obtained from Addis Ababa University to black lion specialized hospitals manager, endocrine clinic, and nuclear medicine department of TASH. Oral communication was sought by phone for patients included in the study, and the result of the study was communicated to patients and responsible bodies for any beneficiary or corrective measures. Confidentiality was kept at each step of data collection and processing.

### Statistical analysis

Excel data sheet and SPSS version 20.0 were used to analyze data obtained from patient’s medical record. The data were expressed as range, mean and standard deviation of the mean, and *P* values. Two-sample *T* test was used to check the association between variables. The level of significance was set at *p* < 0.05. Normality was checked by one-sample *K*-*S* test where *P* value > 0.05.

## Results

A total of 9000 patients included in this study, of which 582 study subjects were identified with subclinical hypothyroidism with a prevalence rate of 6.47%. Their age ranged from 18 to 90 years, with mean age of 38.83 ± 14.65 SD years.

Of ScHt patients, 162 (28.82%) were male and 420 (71.18) % were female. About 55.6% of the study subjects were living in Addis Ababa city while the rest 44.4% were from other regions. The prevalence of ScHt was 4.6% in female and 1.9% in male.

Of the total subclinical hypothyroid patients, most patients were diagnosed with typical symptoms of hypothyroidism or asymptomatic. Most (74%) had serum TSH levels between 5 and 10 mIU/L, and some (26%) had serum TSH level > 10 mIU/L.

### Symptoms

**Fig. 1:**
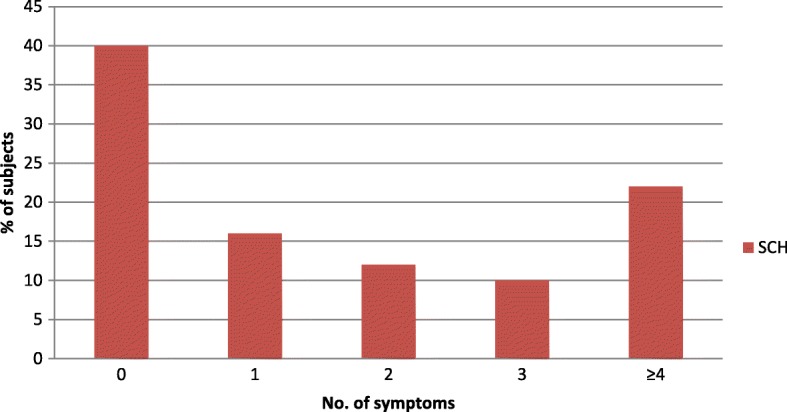
Symptom frequency of subclinical hypothyroid subjects at endocrine clinic of TASH

The percentage of subjects with no symptoms hypothyroidism was 40%, and with 1, 2, 3, and 4, it was 16, 12, 10, and 22% respectively. The proportion of subclinical hypothyroid subjects rose as the number of symptoms increased (Fig. 1). That is, as more symptoms were reported, the subject was more likely to be subclinical hypothyroid.

**Fig. 2:**
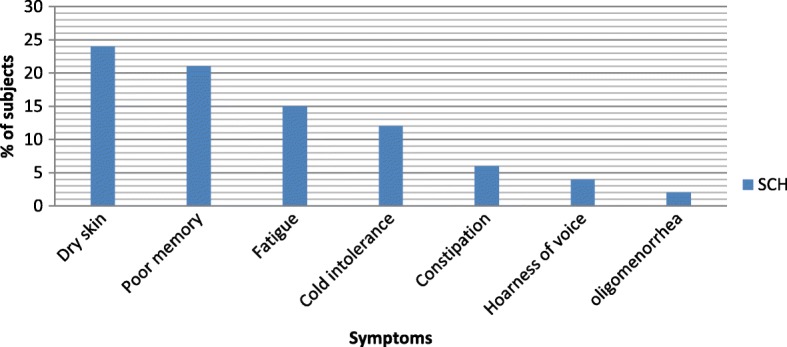
Subclinical thyroid subjects with observed symptoms at endocrine clinic of TASH, 20153

Subjects who were identified as subclinical hypothyroid often reported of having dry skin (24%), poor memory (21%), fatigue (15%), cold intolerance (12%), constipation (6%), and hoarseness of voice (4%) (Fig. 2).

### Age and subclinical hypothyroidism

The average TSH of subclinical hypothyroid patients whose age is ≥ 40 differ significantly from that of subclinical hypothyroid patients whose age is < 40 and it showed increment as shown in Table 1. This study showed there was no significant difference in TT3 between those subclinical hypothyroid patients whose age is ≥ 40 and those whose age is < 40 but the mean TT4 value decreased further with age between the two groups and the difference was statistically significant (Table 1).

**Table 1:** Mean level of thyroid function tests (TFT) and age among subclinical hypothyroid patients in TASH, 2016

TFT	Age	Mean (±SD)	P* value
TSH	≥ 40	11.54(±10.29)	0.015
	< 40	8.71(±6.82)	
TT4	≥ 40	100.79(±21.96)	0.04
	< 40	104.42(±22.34)	
TT3	≥ 40	2.11(±0.57)	0.93
	< 40	2.19(±0.53)	

*Two-sample *T* test

### Sex and subclinical hypothyroidism

The mean serum TT4 found in females was slightly higher than that of male but the difference between the two groups was statistically insignificant. The average TSH and TT3 of subclinical hypothyroid male patients did not differ significantly from that of subclinical hypothyroid female patients as shown in Table 2.

**Table 2:** Mean concentration of thyroid function tests and sex among subclinical hypothyroid subjects in TASH, 2016

TFT	Sex	Mean (±SD)	P* value
TSH	Female	10.88 (±9.53)	0.96
	Male	9.66 (±8.33)	
TT4	Female	103.09 (±22.16)	0.49
	Male	101.48 (±22.40)	
TT3	Female	2.13 (±0.55)	0.12
	Male	2.21 (±0.55)	

*Two-sample *T* test

The mean serum TT3 and TT4 of female whose age is between ≥ 40 and < 40 did not show significant difference whereas the average TSH of subclinical hypothyroid female patients whose age is ≥ 40 differ significantly from that of subclinical hypothyroid female patients whose age is < 40. However, the mean TSH showed increment and that of TT4 were decreased as shown in Table 3.

**Table 3:** Mean concentration of thyroid function tests among female subclinical hypothyroid patients in TASH, 2016

Thyroid function test	Age	Mean (±SD)	P* value
TSH	≥ 40	11.46 (±10.38)	0.048
	< 40	7.93 (±5.19)	
TT4	≥ 40	101.24 (±22.17)	0.07
	< 40	105.21 (±21.98)	
TT3	≥ 40	2.11 (±0.55)	0.51
	< 40	2.15 (±0.55)	

*Two-sample *T* test

There was no significant difference, between male whose age is between ≥ 40 and < 40, in TSH, TT4, and TT3. However, the mean TT4 decreases with age as shown in Table 4.

**Table 4:** Mean concentration of thyroid function tests among male subclinical hypothyroid patients in TASH, 2016

Thyroid function tests	Age	Mean (±SD)	P* value
TSH	≥ 40	11.79 (±10.12)	0.14
	< 40	10.31 (±9.14)	
TT4	≥ 40	98.39 (±21.39)	0.26
	< 40	102.86 (±23.05)	
TT3	≥ 40	2.12 (±0.61)	0.08
	< 40	2.27 (±0.49)	

*Two-sample *T* test

## Discussion

In this study, the prevalence of subclinical hypothyroidism was 6.47%. Similar results of prevalence of ScHt have been reported to be between 3 and 12% of adult population worldwide [3–13], and in harmony with our study, a prevalence rate of 6.31% was reported in India having TSH value in between 4.94 and 10 μIU/ml. In contrast to this study, prevalence of ScHt 19.3% [23] was recorded in another study in India. These differences could be explained by iodized salt supplementation given to patients in before the actual study. Subclinical hypothyroidism is more prevalent in iodine-rich areas, 6.1 to 18.0% compared with 0.9 to 3.8% in iodine-deficient areas [12]. Even though Addis Ababa is an iodine-deficient area, a higher prevalence of ScHt among the participants may need further investigation.

Higher prevalence of ScHt among females in the current study is in line with the findings in the Wickham survey [6] and according to a study conducted in India [24], Northern Europe [25], and Framingham study [26]. Even though higher prevalence of ScHt in women is unclear, it could be due to estrogen effect, higher prevalence of autoimmune thyroid diseases, and higher concentration of thyroperoxidase antibodies and thyroglobulin antibodies in females.

The incidence of ScHt was significantly lower in the age group between 19 and 40 years for both male and female sexes. In harmony with our finding, significantly higher TSH value was obtained in an Indian study [27] and Busselton Health survey [28]. Similar findings were also observed in US population in which TSH level showed higher level among age group ≥ 40 years old where the mean value was 7.49 mIU/L as compared with 8.71 mIU/L in the current study [29]. An age-related TSH increase could be explained by a normal physiologic response to compensate for the decrease in TSH biological activity due to age-related changes in TSH glycosylation or decreased thyroxine turnover with aging. Decreased sensitivity of the thyroid gland to TSH and increased prevalence of thyroid autoantibodies with aging is also a possible mechanism.

In this study it is found that most 74% of them had serum TSH levels between 5 and 10 mIU/L. Our finding was corroborated with studies conducted abroad where 74 to 87% of the participants were having serum TSH levels between 5 and 10 mIU/L [8, 19, 30].

A growing body of evidences reported that higher TSH and prevalence rates for hypothyroidism in women and with advancing age [31–36] with rates as high as 24% among women older than 60 years recruited from several senior citizens’ centers and ambulatory clinics [27].

In this study, subjects who were identified as subclinical hypothyroid more often reported having dry skin (24%), poor memory (21%), and fatigue (15%) as it was observed in studies conducted elsewhere [8, 24, 32–35]. A small increase in total symptoms was observed with progressive deterioration of thyroid function. Moreover, reporting more symptoms, in particular recently “changed symptoms” increased the likelihood of disease.

## Conclusions

Overall prevalence of subclinical hypothyroidism observed in our study was higher. Older age groups were having a significant difference serum TSH level than other age groups. Women become more vulnerable to develop ScHt than males. Almost 25% of the study participants developing ScHt were having four symptoms. Screening and concern should be given for patient developing ScHt because they may develop overt hypothyroidism which could have serious complication.

### Limitation of the study

The study is a retrospective study where the source of information is secondary data, and a substantial proportion of patients were excluded for incomplete medical records. There are no published literatures in the country on ScHt as per the investigators knowledge which makes our study difficult to compare and progressive outcome of patients from subclinical hypothyroidism to overt hypothyroidism was not assessed. The study is limited to findings in a single hospital, and detailed patient characteristics were not assessed.

## Abbreviations

Ab: Antibody
Ag: Antigen
IU: International unit
rpm: Revolution per minute
ScHt: Subclinical hypothyroidism
SD: Standard deviation
TASH: Tikur Anbesa Specialized Hospital
TSH: Thyroid-stimulating hormone
TT_3_: Triiodothyronine
TT_4_: Total thyroxine
